# Impact of Latent Virus Infection in the Cornea on Corneal Healing after Small Incision Lenticule Extraction

**DOI:** 10.3390/microorganisms11102441

**Published:** 2023-09-28

**Authors:** Ming Liu, Wenting Song, Wen Gao, Lili Jiang, Hongbiao Pan, Dan Luo, Lei Shi

**Affiliations:** 1 Department of Ophthalmology, Anhui Second Provincial People’s Hospital, Dangshan Road 1868, Hefei 230041, Chinagwahyk@126.com (W.G.); 18110929750@163.com (L.J.); 13855110088@163.com (H.P.); 2 Department of Ophthalmology, The First Affiliated Hospital of USTC, Hefei 230001, China; poppyvita@163.com (W.S.); ldzg2013@sina.cn (D.L.)

**Keywords:** lenticule, femtosecond laser small incision lenticule extraction, virus infection, corneal healing

## Abstract

The aim of the present study is to analyze the impact of cornea virus latent infection on corneal healing after small incision lenticule extraction (SMILE) and predict the positive rate of virus latent infection in corneal stroma. A total of 279 patients who underwent SMILE were included in this study. Fluorescence quantitative PCR was used to detect virus infection in the lenticules, which were taken from the corneal stroma during SMILE. Herpes simplex virus type 1 (HSV-1), herpes simplex virus type 2 (HSV-2), Epstein–Barr virus (EBV), and cytomegalovirus (CMV) were detected. Postoperative visual acuity, spherical equivalent, intraocular pressure, corneal curvature (Kf and Ks), corneal transparency, and corneal staining were compared between the virus-positive group and the virus-negative group. The number of corneal stromal cells and inflammatory cells, corneal nerve fiber density (CNFD), corneal nerve branch density (CNBD), corneal nerve fiber length (CNFL), corneal total branch density (CTBD), and corneal nerve fiber width (CNFW) were evaluated using an in vivo confocal microscope. Out of 240 herpes simplex virus (HSV) tested samples, 11 (4.58%) were positive, among which 5 (2.08%) were HSV-1-positive and 6 (2.50%) were HSV-2-positive. None of the 91 CMV- and EBV-tested samples were positive. There was no statistical significance in the postoperative visual acuity, spherical equivalent, intraocular pressure, Kf and Ks, corneal transparency, corneal staining, the number of corneal stromal cells and inflammatory cells, CNFD, CNBD, CNFL, CTBD, and CNFW between the virus-positive and virus-negative groups (*p* > 0.05). In conclusion, there is a certain proportion of latent HSV infection in the myopia population. Femtosecond lasers are less likely to activate a latent infection of HSV in the cornea. The latent infection of HSV has no significant impact on corneal healing after SMILE.

## 1. Introduction

The prevalence of viral keratitis is very high, and there are around 10 million people with viral keratitis worldwide. It is the leading cause of corneal opacity and infectious blindness [1,2]. The high recurrence rate of viral keratitis is related to the latent infection of the virus. The latency and reactivation of the virus are the result of the interaction between the virus and host factors [3]. Stress, ultraviolet light, heat, hormones, or excimer lasers can also trigger the reactivation of latently infected viruses. Several studies have confirmed the latent infection and persistence of viruses in corneal tissues [3], including herpes simplex virus type 1 (HSV-1), herpes simplex virus type 2 (HSV-2), cytomegalovirus (CMV), Epstein–Barr virus (EBV), varicella-zoster virus (VZV), etc. [2]. With the development of laser technology, corneal refractive surgery is widely recognized for its high safety and efficacy [4,5]. However, there were still some case reports about viral keratitis that happened after corneal refractive surgery using femtosecond lasers [6,7,8]. Until now, whether a femtosecond laser could activate the latent virous infection remains unclear. Therefore, we plan to explore whether the laser caused viral activation in the corneal stroma to induce viral keratitis. With the surgery of femtosecond laser small incision lenticule extraction (SMILE), a lenticule can be extracted from the corneal stroma, which can be used for virus detection. Besides, poor corneal healing is one of the problems after SMILE [9]. Confocal microscopy can be used to scan different layers of living cornea and observe the cells and nerve fibers of each layer of cornea, which can be used to evaluate corneal healing after refractive surgery [10,11]. The purpose of this study is to detect the virus infection of lenticules and evaluate the healing process in patients with or without virus infection.

## 2. Materials and Methods

### 2.1. Participants

The study strictly followed the tenets of the Declaration of Helsinki and was approved by the Medical Ethical Committee of the First Affiliated Hospital of USTC (No. 2023RE006). A total of 279 patients who underwent SMILE for refractive error correction in the First Affiliated Hospital of USTC from January 2022 to October 2022 were included.

### 2.2. Inclusion Criteria

Patients in a good psychological state with a clear intention to operate, aged between 18 and 45 years old, were included in the study. The equivalent spherical lens was in the range of −1.50D to −8.00D. They met the surgical indications and had no other eye diseases.

### 2.3. Exclusion Criteria

Patients had diseases that affected postoperative recovery, as well as intraoperative desorption and poor lens separation.

### 2.4. Examinations

All patients underwent comprehensive preoperative examinations, including slit-lamp and fundus examinations, uncorrected and corrected distance visual acuity, spherical equivalent refraction, intraocular pressure, an ophthalmic optical biometer (IOL Master, Zeiss, Oberkochen, Germany), and an anterior segment analyzer (Pentacam 70700, Oculus, Wetzlar, Germany).

### 2.5. Surgical Procedures

SMILE surgery was performed by three experienced physicians using the VisuMax femtosecond laser system (Carl Zeiss Meditec AG, Jena, Germany). After disinfection and topical anesthesia, the eyeball was fixed by a negative pressure suction ring. Laser scanning was started after confirmation of accurate positioning. The pulse frequency was set to 500 kHz. The incision was located at 12 o’clock and was 2 mm wide. Then, the lenticules were separated and obtained from the incision. Lenticules were placed in a sterilized Eppendorf tube, then stored at −80 °C for further examinations in 1 month.

### 2.6. Virus Detection

Viruses were detected with HSV-1, HSV-2, CMV, and EBV fluorescence quantitative polymerase chain reaction (FQ-PCR) kits (ABI 7500, USA) following the manufacturer’s instructions.

The results were stored and analyzed automatically after the reaction. The results were recorded as negative when the sample amplification curve was not S-shaped, and the cycle threshold value was UNDET or 40.00. Retesting was recommended when the cycle threshold value was between 37.00 and 40.00. The results were recorded as positive when the sample had an S-shaped amplification curve and a cycle threshold value ≤ 37.00.

### 2.7. Postoperative Examinations

Visual acuity, intraocular pressure, and spherical equivalent refraction were examined. And all patients were examined using slit lamp microscope and Pentacam.were examined using a Pentacam slit lamp microscope on day 1 and at 1 week and 1 month after surgery. Corneal transparency (transparency, edema) and corneal fluorescent staining (non-staining, staining) were recorded. All eyes of the virus-positive group and eyes randomly selected from the control group underwent corneal confocal microscopy examination 1 month after surgery.

### 2.8. Corneal Confocal Microscopy

An examination using the corneal confocal microscope HRT3 (Heidelberg Company, Germany) was performed at the center of the cornea along the sagittal axis by the same experienced physician. One image was selected from each shallow stromal layer, middle stromal layer, and deep stromal layer. The number of stromal cells was counted using Image J software (National Institutes of Health, America). Two images with visible inflammatory cells were selected from each eye, and the number of cells was counted to calculate the average value. Three clear images of the nerve fiber layer were chosen from each eye and quantified using ACC Metrics (a nerve fiber analysis software from the University of Manchester). The analyzed parameters included: corneal nerve fiber density (CNFD): number of nerve fibers/mm^2^; corneal nerve branch density (CNBD): number of nerve branches/mm^2^; corneal nerve fiber length (CNFL): length of nerve fibers (mm)/mm^2^; corneal total branch density (CTBD): number of all nerve branch nodes/mm^2^; and corneal nerve fiber width (CNFW): the average width of nerve fibers (mm).

### 2.9. Statistical Analysis

Prism 9.3 statistical software was used to analyze the data. The measurement samples were expressed as the mean ± standard deviation (SD). The two groups of measurement samples were tested by an independent sample *t*-test if they conformed to a normal distribution and homogeneity of variance and a Mann–Whitney test if they did not. Count data were expressed as frequency and compared by the chi-square test. *p* < 0.05 was considered a significant difference.

## 3. Results

### 3.1. Results

#### 3.1.1. General Results

A total of 11 of 240 samples were HSV-positive (4.58%), including 5 HSV-1-positive (2.08%) and 6 HSV-2-positive cases (2.50%). A total of 91 samples were tested for EBV and CMV, and all of them were negative.

There were no significant differences in gender, age, preoperative spherical equivalent, preoperative corrected visual acuity, preoperative corneal curvature (Kf and Ks), preoperative corneal thickness, preoperative intraocular pressure, axial length, optical zone diameter, lenticule depth, residual corneal thickness, or added refraction value between HSV positive and negative groups (*p* > 0.05) (Table 1).

There were also no significant differences in postoperative uncorrected visual acuity, spherical equivalent, intraocular pressure, corneal curvature (Kf and Ks), corneal thickness, corneal transparency, or corneal fluorescein sodium staining between HSV-positive and HSV-negative groups (*p* > 0.05) (Table 2).

#### 3.1.2. Corneal Confocal Microscopy Results

All 11 HSV-positive patients (21 eyes) underwent confocal microscopy on the cornea, as well as 15 randomly selected HSV-negative patients (15 eyes).

**Figure 1:**
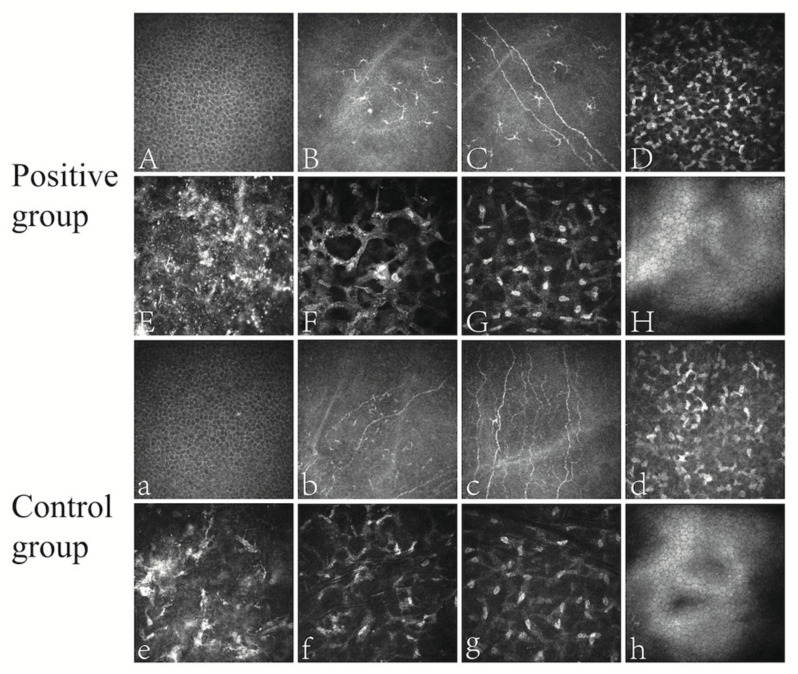
Corneal confocal microscopy in the positive and control groups after SMILE. Positive group: (**A**) corneal epithelial layer; (**B**) epithelial inflammatory cells; (**C**) nerve fibers and surrounding dendritic cells; (**D**) superficial corneal stroma; (**E**) hyperreflective debris of the surgical interface; (**F**), activated stromal cells in the shape of polygons or crab claws; (**G**) deep corneal stroma; (**H**) endothelial surface. Control group: (**a**) corneal epithelial layer; (**b**) nerve fibers and surrounding inflammatory cells; (**c**), nerve fibers; (**d**), superficial corneal stroma; (**e**) hyperreflective material at the surgical interface; (**f**) activated corneal stroma; (**g**) deep corneal stroma; (**h**) endothelial surface.

Both groups presented regular epithelial cells (Figure 1A,a) under confocal microcopy. Inflammatory cells such as dendritic cells were visible in the epithelial and superficial stromal layers (Figure 1B,b). Corneal nerve fibers were easy to recognize (Figure 1C,c). There were highly reflective and dense stromal cells on top of the surgical interface (Figure 1D,d), while debris-like reflection was found in the interface (Figure 1E,e). Beneath the interface, there were polygonal or crab-claw-shaped activated stromal cells (Figure 1F,f). The density of stromal cells gradually decreased from the superficial to the deep corneal layer (Figure 1G,g). Endothelial cells were regular with hyper-reflective cytoplasm (Figure 1H,h).

The images of confocal microscopy were quantified using Image J and ACC Metrics software. There were no significant differences in the number of corneal stromal cells, inflammatory cells, CNFD, CNBD, CNFL, CTBD, or CNFW between the HSV-positive group and HSV-negative group (*p* > 0.05) (Table 3).

## 4. Discussion

The high incidence and recurrence rate of viral keratitis are related to the infectious type. The infection is a lifelong persistent or latent infection [12]. Most humans probably experience oral HSV infection, with subsequent viral spreading to the trigeminal ganglia. Viral replication may occur in the trigeminal ganglia until CD8+ T-cell activity. The viruses are probably driven into latency due to the inefficiency of the DNA repair mechanism [13]. In addition to sensory neurons, aqueous humor, and the iris, the cornea is also one of the sites of latent infection and persistence [3]. Reactivation may be triggered by several factors, including stress, UV radiation, and fever. Most of the keratitis morbidity associated with HSV is observed in the recurrences of latent infection rather than the initial ocular infection [14,15,16]. Herpes simplex keratitis (HSK) can be further classified anatomically into epithelial, stromal, or endothelial forms. Stromal HSK accounts for only 2% of the initial ocular manifestations of HSV but causes 20% to 61% of recurrent disease. Young et al. [17] found that the risk of ocular HSV recurrence was 27%, 50%, and 57% at 1, 5, and 10 years, respectively, but if it recurred immediately after the initial attack, the risk of recurrence increased to 38% and 67% at 1 and 5 years, respectively.

With the development of laser technology, the acceptance of corneal refractive surgery is increasing. With a small incision, a lenticule could be extracted during SMILE. The thickness of lenticules routinely varies from tens to more than 100 μm according to the degree of myopia and astigmatism. These lenticules can be high-quality corneal donors for the treatment of keratoconus, corneoscleral dermoids, corneal ulcers, and other corneal diseases. These corneal stromal lenticules are a new source of collagen-rich extracellular matrix scaffolds, which might be a supplement to the corneal donor shortage [18,19]. Qu et al. [20] evaluated the clinical outcomes of keratoplasty with virus-positive grafts and found that HSV-1 can be transmitted from the graft to the recipient. Therefore, HSV-positive donor corneas cannot be an ideal donor for keratoplasty. This provides a great advantage for the detection of latent virus infection in the cornea.

In this study, FQ-PCR was used to detect the latent virus infection in the cornea to further confirm the persistence of the related virus in the cornea and analyze the positive rate of latent virus infection in the normal population.

A total of 240 HSV samples were tested in this study, and 11 cases were HSV-positive, including 5 HSV-1-positive cases, 6 HSV-2-positive cases, and 229 HSV-negative cases. Ninety-one cases of EBV and 91 cases of CMV were tested, and all of them were negative. The positive rate of HSV was 4.58%, 2.08% for HSV-1, and 2.50% for HSV-2. There are few reports on the detection of viruses in lenticules. Shang et al. [21] used FQ-PCR technology to detect 128 corneal stromal lenticule samples from 64 patients, and both HSV-1 and HSV-2 were negative. There are more reports on the detection of viruses in donated corneas. Qu et al. [20] detected HSV-1, HSV-2, VZV, CMV, and EBV DNA in 942 donated corneas, and 23 of them (2.44%) were positive for herpesvirus DNA. The positive rates of HSV-1, CMV, VZV, and EBV were 0.74%, 0.85%, 0.64%, and 0.21%, respectively. This is somewhat different from the results of our study, which may be related to the differences in the age composition of the enrolled study subjects, sample preservation, testing procedures, or sample sources. Qu et al. [20] showed that the virus detection rate was different in different age groups; the detection rate of patients aged under 50 years old (4.55%) was higher than that of patients aged over 50 years old (3.07%). However, the age range of our study was 18 to 45. Therefore, there may be differences in the detection rate. In our study, EBV and CMV were not detected, which were different from 0.21% and 0.85% positive rates, respectively, as reported in the study of Qu et al. [20]. This may be related to the site of latent virus infection in the cornea. In our study, the lenticules removed during SMILE were mainly from the superficial stroma, while the detection of the donated cornea could involve the whole corneal layer. Therefore, it is possible that the latent infection sites of EBV and CMV in the cornea may be located in other parts of the cornea. The specific sites of latent infection of herpes viruses, including HSV, in the cornea deserve further study.

Shang et al. [21] found a low risk of infection with SMILE corneal stromal lenses, but there is a risk of rejection with fresh stromal lenses. They found anhydrous glycerol preservation at −78 °C is an ideal way to reduce antigens without compromising the structure and function of the lens. Our study successfully detected a latent infection of HSV in the corneal stroma. Therefore, with the increasing application of the corneal lenticules, we need to pay more attention to sample preservation and infection transmission during their application.

Infectious keratitis is one of the potentially serious complications after refractive surgery. Even though it decreased in the 2000s, it still occurs at 0.02% to 0.2% [22]. There have been few case reports of herpetic ophthalmopathy after SMILE. But animal studies have shown that PRK and LASIK can cause the reactivation of latent HSV [23,24,25], and studies showed that after surgery with a keratome or excimer laser, reactivation may occur even in the absence of prior herpetic virus disease [7,23,25]. SMILE theoretically causes less damage to the corneal epithelium than other refractive surgeries, but it may trigger the reactivation of latent HSV. The exact mechanism of virus reactivation caused by excimer lasers is unknown. Theoretically, it may be due to the death of neuronal cells [6]. Steroid eye drops can be routinely applied after corneal refractive surgery to reduce corneal inflammation. Studies have shown that steroid hormones can reduce the number of immune cells and the binding ability of immunoglobulins to cell surface receptors and inhibit the synthesis and release of immune factors. These could cause immunosuppression and induce virus activation [26].

In our study, HSV was detected in the corneal stromal lenticule. However, there were no differences in the postoperative visual acuity, spherical equivalent, corneal curvature, or corneal thickness between the HSV-positive and HSV-negative groups. The proportion of corneal edema was 3.40%, which was higher than 0.09% in the study of Wang et al. [27]. The proportion of corneal epithelial damage was 1.06%, which was also different from the study of Wang et al. (3.26%) [27]. This may be related to the differences in the sample size of the study (470 eyes) and the statistical sample size of Wang et al. (6373 eyes), the specific conditions of surgery, the source, and the basic characteristics of patients. These results suggest that the latent presence of viruses in corneal tissue has no significant effect on the occurrence of infectious keratitis after SMILE. Since HSV is more latent in the sensory neurons of the human body, it is reasonable to suspect that the viral source of viral keratitis after SMILE is more likely to come from sensory neurons. This is not consistent with the view that postoperative infection after SMILE, like other refractive surgery, is caused by microorganisms in the corneal stromal pocket [28], so this deserves further investigation. With no evidence of postoperative infection in either group and no indication of using antiviral therapy or decreasing hormone therapy, we speculate that, unlike excimer laser, femtosecond laser may not cause or have a weak effect on viral reactivation.

After corneal refractive surgery, epithelial injury can trigger the release of cytokines and growth factors, including interleukin-1 and tumor necrosis factor-α. These cytokines directly induce apoptosis of corneal cells, thus initiating a complex cascade in which the surrounding living corneal cells are activated and transformed into fibroblasts or myofibroblasts. Chemokines attract inflammatory cells, including monocytes and lymphocytes, into the corneal stroma to engulf apoptotic and necrotic debris [29]. Subsequently, myofibroblasts achieve extracellular matrix and stromal remodeling through the production of collagen, glycosaminoglycan, collagenase, gelatin enzyme, and matrix metalloproteinase [30]. Studies have shown that inflammation, apoptosis, and fibrosis are common phenomena in wound healing after corneal refractive surgery [31]. However, compared with LASIK, SMILE induces less apoptosis, proliferation, and inflammation of corneal cells, which may be related to the different working principles of excimer lasers and femtosecond lasers. Excimer lasers use ultraviolet light to break molecular bonds in the corneal matrix. The femtosecond laser is a near-infrared laser that cuts the corneal stroma through photodissociation with less tissue damage.

Corneal confocal microscopy provides an effective tool to evaluate the healing of the cornea from a cytological level [32]. The healing of the cornea after SMILE is a complicated process that is affected by multiple factors, such as age [33]. The age of these two groups showed no significant difference, and its influence could be ruled out.

Under confocal microscopy, stromal and inflammatory cells did not show a significant difference between the HSV-positive and HSV-negative groups. The results indicated that the latent presence of virus in the cornea had no significant effect on the regeneration of stromal cells and did not cause additional inflammatory responses. This finding is consistent with the results showing no significant differences in corneal epithelial damage between the two groups. Epithelial damage often leads to the delay of stromal cell regeneration and causes an inflammatory response. In the study of Li et al. [33], the corneal epithelial wing cells, stromal cells, and endothelial cells before and after SMILE did not differ among different age groups, which is different from Zheng et al. [34], in which they found that the density of corneal epithelial cells and endothelial cells gradually decreased with age through in vivo confocal microscopy, probably due to the age limit of SMILE surgery.

In this study, ACC Metrics software was used to quantitatively process the corneal nerve fibers on confocal microscopy images. The postoperative CNFD, CNBD, CNFL, CTBD, and CNFW of these two groups were basically consistent with the results of Li et al. [33].

The corneal nerve is important since it can provide nutritional support for the cornea and is beneficial to corneal repair [35]. Studies have found that the density of the corneal subbasal nerve decreased after SMILE, and the nerve fibers began to increase significantly from 3 to 6 months after surgery [33]. However, Liu et al. found that the subbasal nerve, including CNFD and CNBD, still decreased 5 years after SMILE [36]. Short-term [37] and long-term [36] studies showed that corneal nerve repair was significantly reduced in the LASIK group, but no significant change was found in the SMILE group. Li et al. [33] studied the effect of age on corneal nerve fiber recovery and found that the CNFL of patients aged younger than 30 years old recovered to preoperative level 1 year after surgery, while the CNFL of patients aged older than 30 years old was shorter than that before surgery. However, the latent presence of virus in the cornea has no significant effect on the healing of corneal nerve fibers after SMILE.

## 5. Conclusions

In conclusion, there is a certain proportion of latent HSV infections in SMILE patients without any symptoms or signs. The specific sites of latent infection of herpes viruses in the cornea deserve further study. We need to pay more attention to sample preservation and infection transmission during its application. Femtosecond lasers are unlikely to activate a latent HSV infection in the cornea. The latent infection of HSV has no significant effect on corneal healing after SMILE without increasing the risk of infectious keratitis, which further confirms the safety of SMILE.

## Figures and Tables

**Table 1:** The general condition of patients in the positive group and the control group.

General Condition	Positive Group (n = 21)	Control Group (n = 449)	*p* Value
Gender (male/female)	6/5	162/67	0.3129
Age (years)	21.73 ± 2.05	22.99 ± 4.79	0.9110
Preoperative spherical equivalent (D)	−5.67 ± 1.37	−4.98 ± 1.64	0.0504
Preoperative corrected visual acuity	0.01 ± 0.06	−0.01 ± 0.07	0.1152
Preoperative corneal curvature, K_f_ (D)	42.46 ± 0.68	42.06 ± 1.19	0.1307
Preoperative corneal curvature, K_s_ (D)	43.84 ± 0.75	43.28 ± 1.35	0.0598
Preoperative corneal thickness (μm)	547.43 ± 16.36	548.34 ± 24.59	0.8447
Preoperative intraocular pressure (mmHg)	18.37 ± 2.27	18.20 ± 2.28	0.9283
Axial length (mm)	25.46 ± 1.01	25.67 ± 0.91	0.3116
Optical zone diameter (mm)	6.68 ± 0.17	6.72 ± 0.14	0.3657
Lenticule depth (μm)	124.14 ± 20.89	116.41 ± 23.57	0.1405
Residual corneal thickness (μm)	305.67 ± 24.10	312.61 ± 26.34	0.2033
Added refractive value (D)	0.83 ± 0.14	0.81 ± 0.14	0.4182

An independent sample *t*-test was used to analyze the differences in preoperative corneal curvature (K_f_ and K_s_), axial length, and lenticule depth between the two groups. The Mann–Whitney test was used to analyze the other indicators. *p* < 0.05 was considered statistically significant. D: diopters.

**Table 2:** The postoperative examination results of patients in the positive group and the control group.

Postoperative Examination Results	Positive Group (n = 21)	Control Group (n = 449)	*p* Value
Postoperative uncorrected visual acuity	−0.02 ± 0.07	0.01 ± 0.09	0.0798
Postoperative spherical equivalent (D)	−0.07 ± 0.45	−0.11 ± 0.52	0.9502
Postoperative intraocular pressure (mmHg)	13.61 ± 0.96	13.19 ± 1.93	0.2793
Postoperative corneal curvature K_f_ (D)	37.95 ± 1.14	38.13 ± 1.71	0.6203
Postoperative corneal curvature K_s_ (D)	38.73 ± 1.21	38.84 ± 1.81	0.7865
Postoperative corneal thickness (μm)	435.57 ± 27.72	444.97 ± 29.15	0.0763
Corneal transparency (edema/transparency)	1/20	15/434	0.5246
Corneal staining (with/without staining)	0/21	5/444	>0.9999

An independent sample *t*-test was used to analyze the differences in postoperative corneal curvature K_f_ and K_s_ between the two groups. The Mann–Whitney test was used to analyze the differences in postoperative uncorrected visual acuity, spherical equivalent, intraocular pressure, and corneal thickness between the two groups. *p* < 0.05 was considered statistically significant. D: diopters.

**Table 3:** Postoperative confocal microscopy results of patients in the positive group and the control group.

	Positive Group (n = 21)	Control Group (n = 15)	*p* Value
Stromal cells number	70.65 ± 30.97	63.71 ± 20.77	0.5536
Inflammatory cells number (<5/5–10/>10)	10/5/6	9/2/4	0.6841
CNFD (/mm^2^)	6.96 ± 8.01	6.53 ± 6.66	0.9306
CNBD (/mm^2^)	4.64 ± 7.75	7.22 ± 11.99	0.4036
CNFL (mm/mm^2^)	7.01 ± 3.52	8.03 ± 3.15	0.0622
CTBD (/mm^2^)	15.32 ± 14.48	18.33 ± 17.85	0.5062
CNFW (mm)	0.0239 ± 0.0032	0.0229 ± 0.0026	0.2346

CNFD: corneal nerve fiber density; CNBD: corneal nerve branch density; CNFL: corneal nerve fiber length; CTBD: corneal total branch density; CNFW: corneal nerve fiber width. The chi-square test was used to analyze the difference in corneal inflammatory cells between the two groups, and the Mann–Whitney test was used to analyze the other indicators. *p* < 0.05 was considered statistically significant.
